# Switching between Phosphorescence and Fluorescence Controlled by Chiral Self‐Assembly

**DOI:** 10.1002/advs.201700021

**Published:** 2017-05-15

**Authors:** Guofeng Liu, Yanli Zhao

**Affiliations:** ^1^ Division of Chemistry and Biological Chemistry School of Physical and Mathematical Sciences Nanyang Technological University 21 Nanyang Link 637371 Singapore; ^2^ School of Materials Science and Engineering Nanyang Technological University 50 Nanyang Avenue 639798 Singapore

**Keywords:** helical structures, mechanoluminescence, phosphorescent‐fluorescent switching, self‐assembly, supramolecular chirality

## Abstract

Helical self‐assembly plays a unique role in regulating the localized excitations of π functional systems, which can also bring highly multi‐scale orders, and show a special effect to tune the energy of electronics, vibration, and rotation of molecules. Due to controllable and dynamic property of chiral self‐assembly, highly ordered and helical assemblies can be obtained to exhibit amplification effect and fascinating photophysical properties in photoluminescence. However, an effective control of singlet‐triplet emissive switching in a unimolecular platform remains a great challenge. Recently, switchable singlet‐triplet emission induced by helical self‐assembly in a unimolecular platform has been developed. By taking advantage of the helical self‐assembly driven by multiple intermolecular hydrogen bonding and strong π‐π stacking interactions, reversible switching between fluorescence and phosphorescence could be efficiently achieved both in *N*,*N*‐dimethylformamide/H_2_O solution and the solid state. The results will inspire the design of other intelligent luminescent materials through chiral self‐assembly and be valuable for interdisciplinary development of supramolecular self‐assembly and related materials science.

Chiral or helical self‐assembly,^1^ inspired by fascinating chiral structures with different scales in biological systems, offers a powerful approach to construct hierarchical and functional nanostructures with intriguing supramolecular chirality that originates from highly ordered stacking of chiral or achiral monomers via noncovalently intermolecular interactions, such as hydrogen bonding, π‐π stacking, and electrostatic interactions. To date, a variety of helical assemblies, constructed from chiral self‐assembly, have been demonstrated to display highly potential applications in catalysis,^2^ sensing and recognition,^3^ chiroptical switches,^4^ chiral templates,^5^ biology,^6^ and circularly polarized luminescence (CPL).^7^ On the other hand, helical self‐assembly may play a unique role in regulating localized excitations of π functional systems, which can bring highly perfect multi‐scale orders and show a special effect to tune the energy of electronics, vibration, and rotation of molecules. In addition, due to controllable and dynamic property of chiral self‐assembly, it is easy to obtain chiral amplification of the aggregates from nanoscale to micron level or even above. The highly ordered and dynamic one‐dimensional helical assemblies may exhibit an amplification effect and possess useful photoelectric functions on corresponding applications, for example, photoluminescence (PL).

PL (including fluorescence and phosphorescence) of organic materials has displayed promising applications in organic light‐emitting diodes (OLEDs), sensors, data storage, and bioimaging.^8^ In the PL process (**Figure** **1**), organic luminogens absorb photons and jump to the excited singlet (S) state. Usually, they return to the ground state through fluorescence or nonradiative transitions in most of luminescent organic materials. In a few cases, they could transit to the excited triplet (T) state through the intersystem crossing (ISC) process and undergo phosphorescent emission, for which, metals or heavy atom effects are generally required.^9^ Compared to inorganic and organometallic counterparts, purely organic molecules are more conveniently designed and their wavelength of emission can be easily tailored. However, room‐temperature phosphorescence (RTP) has only been observed from particular organic molecules, because triplet excitons are readily consumed through nonradiative process. Thus, several feasible strategies have been proposed to promote the ISC process and/or suppress the nonradiative deactivation pathways of triplet excitons. For example, crystallization‐induced phosphorescence (CIP) affords an “engineered crystal” approach to construct efficient purely organic RTP materials.^10^ Meanwhile, noncovalent interactions such as halogen‐hydrogen bonding interactions are also employed to tailor RTP from purely organic materials in amorphous polymer matrices.^11^ For unimolecular luminogens, thanks to different radiation levels of electronic transition, phosphorescent and fluorescent emission may cover a broad range of wavelengths and exhibit dual emissions. Crystallization^12^ and mechanical stimulation^13^ induced fluorescence‐phosphorescence dual emissions have been obtained, respectively. Furthermore, simultaneous occurrence of emission from isolated molecules and excited oligomers in a single luminescent emitter, simultaneously boosted fluorescence and phosphorescence from a single molecule ensemble, and single‐emitting components involving two fluorescence domains corresponding to an excited monomer and an excimer have also been reported.^4^, ^5^, ^6^, ^14^

**Figure 1:**
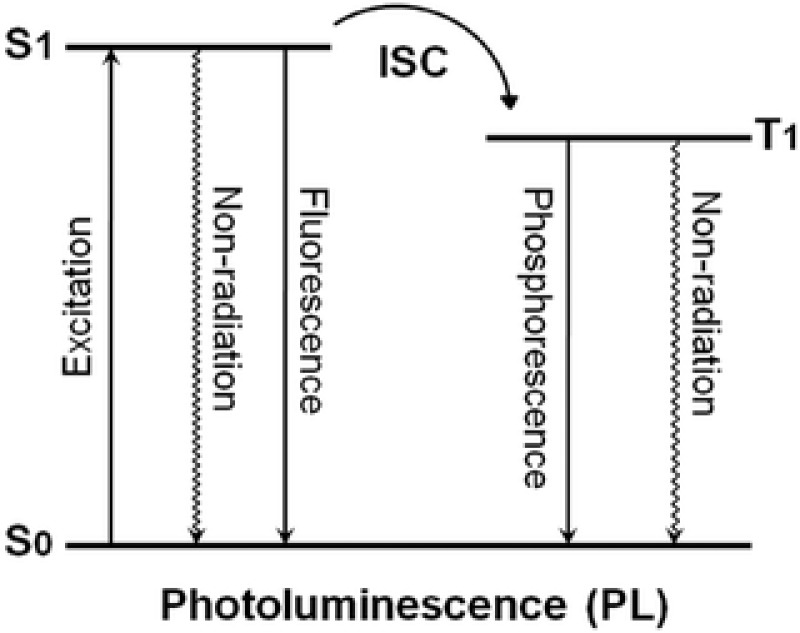
A diagram for photoluminescence including fluorescence and phosphorescence.

Although a significant research progress has been made, the effective control of singlet‐triplet emissive switching in a unimolecular platform remains a great challenge. Recently, Zhu and co‐workers reported a facile approach for switching between fluorescence and phosphorescence by taking advantage of chiral self‐assembly.^15^ In their work, the authors designed and synthesized an asterisk‐shaped hexathiobenzene molecule **1**, which was appended with six (+)‐α‐lipoiate groups by amide bonds at the periphery (**Figure** **2**). Based on the competitive effect of multiple intermolecular hydrogen bonding and strong π‐π stacking interactions driven by chiral lipoiate groups, helical self‐assembly was easily formed, and what is most exciting, the reversible switching between fluorescence and phosphorescence could be efficiently achieved both in *N*,*N*‐dimethylformamide (DMF)/H_2_O solution and the solid state.

**Figure 2:**
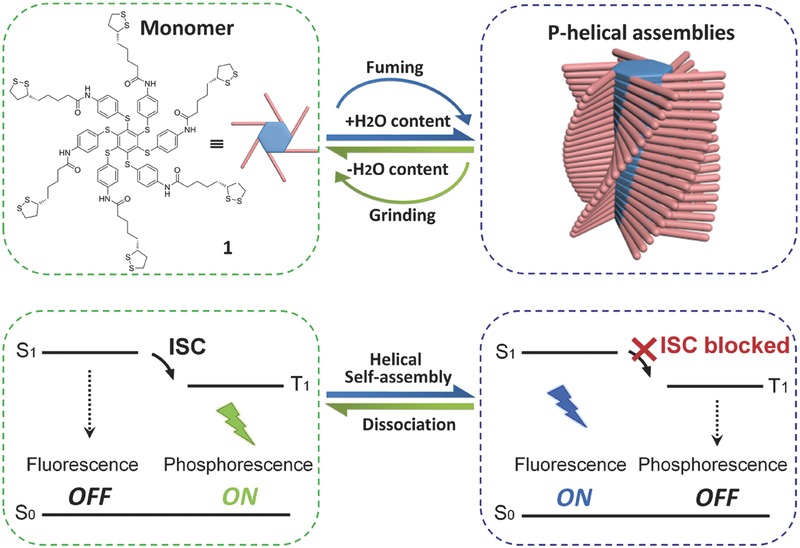
Schematic representation of the structure of monomer **1** and a proposed mechanism of phosphorescence‐to‐fluorescence switching depended on the formation and dissociation of helical self‐assembly.

By employing fluorescence, UV‐Vis and FT‐IR spectroscopy, Zhu and co‐workers demonstrated that the color alteration from green to blue in the mixed DMF/H_2_O solution is attributed to the switching between phosphorescence and fluorescence. Compound **1** displayed a strong blue emission band around 428 nm with the quantum yield of 5.4% in DMF with 30% water, substantially different from a modest green emission at 520 nm with the quantum yield of 1.3% in pure DMF. Such an emission wavelength shift (Δλ > 90 nm) and the difference of quantum yields are remarkable. The result clearly indicates that the luminous process of **1** is highly solvent‐dependent and some unique self‐assembly pathways may play an important role in the emission of aggregate states, since compound **1** has good solubility in DMF but poor solubility in water. Additional evidence for the essence of emissive mechanism during the switching process was obtained by the photoluminescent lifetime measurements. A strong fluorescence emission with a short lifetime of 1.2 ns for the band of 428 nm appeared in DMF with 30% water, compared to a weak phosphorescence band at 550 nm with a relatively long lifetime of 1.3 µs emitted from pure DMF solution.

Transmission electron microscopy (TEM), scanning electronic microscopy (SEM), and circular dichroism (CD) measurements were used to explore the possible self‐assembly pathways and main driving forces inducing the phosphorescence‐to‐fluorescence conversion. From the results of morphologies and CD measurements, helical aggregates started to form in DMF with 20% water and turned to be perfect *P*‐helix when the water fraction reached 30%, accompanied by strong positive‐negative‐positive Cotton effects. These results strongly reveal that the supramolecular chirality of achiral hexathiobenzene core emerges upon the formation of helical self‐assembly, since no CD signal was observed from the achiral hexathiobenzene chromophore in pure DMF solution. In addition, molecular dynamics (MD) simulations provide further insight into the helical superstructures of the helical self‐assembly process. Based on the simulation results, the following self‐assembled processes could be deduced. When exposed to an aqueous environment, the molecules of **1** rapidly aggregate together. Through the synergy between the π‐π stacking of hexathiobenzene cores and the hydrogen bonding interaction of the amide groups unidirectionally driven by chiral lipoiate groups, the layer‐by‐layer hierarchical superstructures form and further assemble into helically advanced architectures with *P* fashion via the extension of a twisting force. In contrast, the achiral reference compounds only displayed a partial triplet‐singlet emission transfer, since they formed less ordered aggregates in the mixed DMF/H_2_O solution.

By utilizing fluorescence and FT‐IR analysis, Zhu and co‐workers demonstrated that the local excited (LE), internal charge transfer (ICT) and π‐π* transitions of **1** in the helical assemblies become discontinuous. Meanwhile, the vibrations of monomer **1** are suppressed by the helical self‐assembly in the aggregated state. Thus, the underlying mechanism of the unique singlet‐triplet emission switching can be proposed as follows (Figure 2). In the monomeric state of **1**, the electrons at ground state (S_0_) absorb photonics and migrate to the singlet states. Due to the overlap between the singlet states and the triplet states, the ISC process is active followed by the phosphorescence decay. Upon the helical self‐assembly, however, the molecular vibrations are discontinuously distributed and the S–T energy gap is enlarged. Consequently, the ISC process is easily blocked to give rise to fluorescence instead of phosphorescence.

In addition, the authors utilized a straightforward technology in the characterization of chiral emissive assemblies. The dynamic nature and environmental sensitivity of supramolecular self‐assembly systems make it difficult to directly verify the relationship between the emissive switching and the supramolecular chirality. Circularly polarized luminescence (CPL) is primarily used to investigate the chiral structures and solution dynamics of luminescent lanthanide complexes. Hence, it can tell chiral information of a molecule in the excited states. The authors employed CPL to thoroughly confirm that the blue fluorescence observed in DMF/H_2_O solution was resulted from helical assemblies, since a CPL signal occurred in mixed DMF/H_2_O solution but was not found in pure DMF. Thus, it can conclude that the helical self‐assembly of **1** triggers the switching of phosphorescence‐to‐fluorescence in DMF/H_2_O solution.

Mechanoluminescence (ML), induced by mechanical stimulation such as grinding, is another important type of luminescence, since mechanical stimulation can be easily imposed on crystal or bulk samples. However, the aggregation caused quenching (ACQ) effect derived from strong π‐π stacking in common organic luminogens could impede the exploration of the ML process. In this work, reversible ML was achieved by grinding and fuming processes. Upon grinding, the luminescent color of helical assemblies was altered from blue to green, accompanied by a remarkable red shift started from 428 nm in the fluorescence spectroscopy. Interestingly, after fuming with the mixed DMF/H_2_O vapor, a fluorescent peak around 428 nm was recovered. On the other hand, the supramolecular chirality disappeared, since the CD signal completely vanished after grinding. Then, CD peaks of the sample were restored after fuming in DMF with 30% water vapor (170 °C, in vacuum). These results reveal that the mechanistic conversion of multicolor luminescence resulted from the disintegration and reformation of the helical aggregates during the grinding and fuming processes are similar to the emissive switching in DMF/H_2_O solution with the variation of water fractions. Thus, the reversible ML of corresponding solid sample of **1** was also controlled by an analogously helical self‐assembly.

The development of advanced functional materials or devices is one of the main stimuli to drive the research of supramolecular self‐assembly. With the rapid development of chiral self‐assembly, chiral electronic or optical devices constructed by supramolecular self‐assembly have attracted increasing interests, since chiral optics and electronics are highly dependent on the properties of helical assemblies. However, it still remains a great challenge to design efficiently molecular systems and establish intelligent optoelectronic devices mediated by helical self‐assembly. The results reported by Zhu and co‐workers can be considered as a significant step forward in the field of controlling phosphorescence‐to‐fluorescence switching in hexathiobenzene‐based unimolecular platforms by helical self‐assembly. The results will inspire the design of other intelligent luminescent materials through chiral self‐assembly. Furthermore, we are convinced that this exploratory research will be valuable for interdisciplinary development of supramolecular self‐assembly and related materials science.

## References

[1] a) D. K. Smith , Chem. Soc. Rev. 2009, 38, 684;19322462

[2] a) L. Qin , L. Zhang , Q. Jin , J. Zhang , B. Han , M. Liu , Angew. Chem. Int. Ed. 2013, 52, 7761; 10.1002/anie.201302662 23776072

[3] a) X. Chen , Z. Huang , S. Y. Chen , K. Li , X. Q. Yu , L. Pu , J. Am. Chem. Soc. 2010, 132, 7297; 20446686 10.1021/ja102480t

[4] Z. G. Zheng , Y. Li , H. K. Bisoyi , L. Wang , T. J. Bunning , Q. Li , Nature 2016, 531, 352. 26950601 10.1038/nature17141

[5] a) J. H. Jung , H. Kobayashi , M. Masuda , T. Shimizu , S. Shinkai , J. Am. Chem. Soc. 2001, 123, 8785; 11535084 10.1021/ja010508h

[6] a) R. K. Das , O. F. Zouani , C. Labrugère , R. Oda , M. C. Durrieu , ACS Nano 2013, 7, 3351; 23451935 10.1021/nn4001325

[7] Z. C. Shen , T. Y. Wang , L. Shi , Z. Y. Tang , M. H. Liu , Chem. Sci. 2015, 6, 4267. 10.1039/c5sc01056j PMC5707475 29218194

[8] a) J. Kuijt , F. Ariese , U. A. T. Brinkman , C. Gooijer , Anal. Chim. Acta. 2003, 488, 135;

[9] a) S. Hirata , K. Totani , J. Zhang , T. Yamashita , H. Kaji , S. R. Marder , T. Watanabe , C. Adachi , Adv. Funct. Mater. 2013, 23, 3386;

[10] a) O. Bolton , K. Lee , H. J. Kim , K. Y. Lin , J. Kim , Nat. Chem. 2011, 3, 205; 21336325 10.1038/nchem.984

[11] a) D. Lee , O. Bolton , B. C. Kim , J. H. Youk , S. Takayama , J. Kim , J. Am. Chem. Soc. 2013, 135, 6325; 23521108 10.1021/ja401769g

[12] Y. Gong , L. Zhao , Q. Peng , D. Fan , W. Z. Yuan , Y. Zhang , B. Z. Tang , Chem. Sci. 2015, 6, 4438. 10.1039/c5sc00253b PMC5665162 29142698

[13] J. Yang , Z. Ren , Z. Xie , Y. Liu , C. Wang , Y. Xie , Q. Peng , B. Xu , W. Tian , F. Zhang , Z. Chi , Q. Li , Z. Li , Angew. Chem. Int. Ed. 2017, 56, 880. 10.1002/anie.201610453 27936297

[14] a) Y. Liu , M. Nishiura , Y. Wang , Z. Hou , J. Am. Chem. Soc. 2006, 128, 5592; 16637599 10.1021/ja058188f

[15] H. Wu , Y. Zhou , L. Yin , C. Hang , X. Li , H. Ågren , T. Yi , Q. Zhang , L. Zhu , J. Am. Chem. Soc. 2017, 139, 785. 28027639 10.1021/jacs.6b10550

